# Basaglia: the psychiatrist as intellectual, theoretician, and activist for a dialogic citizen psychiatry

**DOI:** 10.1007/s00127-026-03068-6

**Published:** 2026-03-02

**Authors:** Thomas Becker, Luciana Degano-Kieser, Giovanni Stanghellini

**Affiliations:** 1 Department of Psychiatry and Psychotherapy, University of Leipzig Medical Center, Semmelweisstraße 10, O4103 Leipzig, Germany; 2 Zentrum Überleben, Berlin, Germany; 3 Department of Health Sciences, University of Florence, Florence, Italy; 4 Centro de Estudios de Fenomenologia y Psiquiatria, Universidad ′Diego Portales′, Santiago, Chile

**Keywords:** Co-production, Franco basaglia, Italy, Phenomenology, Philosophy, Psychiatric reform

## Abstract

**Purpose:**

Franco Basaglia (1924–1980) was a psychiatrist who played an important role in transforming mental health care in Italy during the 1960s and 1970s. Current mental health debates focus on community care, recovery, and co-production. Against this background, the paper describes theoretical references used by Basaglia and colleagues to psychiatric phenomenology, Jean-Paul Sartre, and Michel Foucault, and the role of psychiatrists as professionals and intellectuals, with a view to their relevance to current mental health care issues.

**Methods:**

The paper is based on a non-systematic search and meta-narrative review of sources in Italian, French. and German, including the Archivio Basaglia in Venice, Italy.

**Results:**

Basaglia and colleagues were influenced by phenomenology, Sartre, and Foucault. The tradition of psychiatric phenomenology helped in focusing on the ‘person’, the ‘body’, and the deprivation of individual rights among mental hospital patients. Sartre’s concepts of a ‘personal ontology of freedom’ evolving towards collective action found an echo in Foucault’s archaeology of madness which highlighted the societal construction of madness and emphasized the knowledge-power link in professional practice. Basaglia insisted on linking theory to anti-institutional work and psychiatric reform practice.

**Conclusion:**

Basaglia’s thinking helped mobilize forces toward reform, and his focus on co-production of psychiatric knowledge with experts by experience was ahead of its time. This could help sharpen the concept of recovery during the current crisis of European mental health reform, moving in the direction of more pronounced attention towards the (non-market) economic concepts of social choice and common goods, and towards more user-controlled services.

## Introduction

In his book ‚The Order of Things‘, Michel Foucault wrote that the way science and societal knowledge had evolved from the 18th to the 20th century might not continue in a straight line [1]. Franco Basaglia (1924–1980), the charismatic and effective Italian psychiatric reformer, was aware of the finite nature of human history. Against the background of mental hospital reality in 1960 s Italy and with a view to philosophy and social science theory, he reflected on the professional responsibilities of psychiatrists as intellectuals, technicians and practitioners and used theoretical reasoning in his work. The reform of psychiatric services in Italy achieved radical change in services including mental hospital closure, a new mental health law (1978), a nation-wide system of community mental health services, inpatient treatment in small general hospital psychiatric units, the closure of forensic mental hospitals, the introduction of smaller residential forensic services, and a low level of coercion. There have been recent reviews of the reform [2, 3]. There was strong leadership in this reform movement with an outstanding role of Basaglia and a group of collaborators [4].

Current psychiatric opinion and guidance emphasize community care, peer involvement, co-production and recovery. These models are championed by WHO and supported by evidence presented by global mental health research [5, 6]. There is also a focus on the legal position of people with mental illness and their rights as citizens and consumers which should always be respected. It has been suggested that both social welfare theory and neoliberal thinking impinge on mental health care guidance and practice, and that the recovery concept originates both in anti-institutional critique and neoliberal thought [7]. Internationally, the community mental health care model holds a strong position across high-, medium and low-resource settings [5, 6]. Levels of implementation differ widely. Thus, the endorsement of user involvement, peer support, co-production of mental health and recovery-focused care is strong. Concurrently, some user movement activists, researchers and mad studies scholars have moved away from the focus on recovery, recovery-oriented care and survivor involvement in services and service development, pointing to issues of conceptual ambiguity or incompatibility [8, 9].

Against this background, the present paper explores theoretical positions that Franco Basaglia and colleagues referred to in their books, articles, talks, and correspondence in discussing the role of psychiatrists (as professionals, intellectuals, and activists) in a period of change in Italian psychiatry and society as a whole. Professional roles with a widening of perspective to the various mental health professions, were discussed [10]. Two texts, using English-language sources [11, 12], have previously discussed the relevance of Antonio Gramsci to the understanding of Basaglia’s work. The present paper explores the impact of phenomenological philosophy and psychiatric phenomenology, and of the thinking of Jean-Paul Sartre and Michel Foucault on Basaglia as reflected in his writings, correspondence, and actions. This review is selective, and given the wealth of material on Basaglia, other influences (e.g., Gilles Deleuze and Félix Guattari) will be dealt with elsewhere. The paper rests on the reading of a range of books, papers and correspondence in Italian, French, and German. The decision to study the impact of phenomenology, Sartre and Foucault and the role of professionals, rests on Basaglia’s roots in psychiatric phenomenology, on his repeated reference to Sartre’s and Foucault’s work, and on the important role of both thinkers in critical debates in Western Europe and America between the 1960s and 1980s. In Sartre’s and Foucault’s writings, common ground can be identified in the emphasis on historical reflection, critique of norms and power relationships, in their dealing with ethical issues, in their contributions to the themes of individual and collective action, and in their discursive approach to reality [13].

We discuss how Basaglia’s theoretical positions and actions relate to the threefold traditions of phenomenology, Sartre’s existentialism and Foucauldian poststructuralism, with a particular focus on issues of professional responsibility. Our focus is on the psychiatric profession, bearing in mind that members of other mental health professions are indispensable in multidisciplinary mental health care. The paper concludes with a discussion of the role of such interactions between theory and practice of the 1960s and 70 s in Italy, and of the question whether such theoretical issues have any bearing on the challenges that psychiatry, as a profession, science and mental health care practice, is facing today.

### Methodology

Themes were studied by adopting a meta-narrative review method. We refer to diverse types of books and texts. In addition, one of the authors (LDK) consulted the Archivio Franco e Franca Basaglia (BA), Serie 11: Corrispondenza 1953–1980, in Venice, Italy, from July to October 2024. The exploration of Franco Basaglia’s archived materials documents his involvement in correspondence in various theoretical fields, including philosophy, psychopathology, social sciences, and politics, and the use of thoughts and concepts from these exchanges in his professional practice.

Basaglia’s use of theoretical references was analysed against the background of his academic work and his farewell to academia. After 12 years of academic psychiatric work at the University of Padua (1949–1961), he left the university and became medical director at the Provincial Mental Hospital in Gorizia in North East Italy (1961). Subsequently, he was a lecturer in mental hygiene at the University of Parma and offered a professorial post in geriatric neuropsychiatry at the University of Pavia in 1976, a post that he declined. Following his appointment as mental hospital director, Basaglia’s thinking and action was stimulated by the desolate reality of people hospitalized in psychiatric hospitals in Italy. He continued to elaborate the phenomenological-existential perspective (see e.g. [14–17]), in parallel with his psychiatric institutional work in Gorizia and Trieste (the post of director of San Giovanni Hospital in Trieste being a subsequent appointment as of 1971) by participating in scientific debates, editing books and writing articles with Franca Ongaro and others. Basaglia was in direct or epistolary contact with a number of well-known intellectuals of his time. He selected themes for his writing and theoretical debates as they appeared relevant to his reform work.

We will refer to a selection of key references regarding the impact and influence of phenomenological philosophy and psychiatric phenomenology, of Sartre’s writings, and of the work of Michel Foucault. Among the sources used are monographic texts, essays and articles on the role of the psychiatrist (as professional, activist and intellectual) of the 1960 s and 1970s.

## Results

### Phenomenology

Phenomenological thinking has been important in psychiatric reform across Europe (and elsewhere). In particular, it contributed to a person-centered perspective in dealing with the experience of (and care for) people suffering from mental illnesses [18–20]. The tradition of phenomenology was available to Basaglia when he moved to his new role.

In a two-part book, Novello [15] deals with the influence of phenomenological psychopathology on Basaglia’s work, focusing on papers published by Basaglia during his tenure at the University of Padua. Novello, who worked in Basaglia’s team in Trieste, describes how some team discussions referred to texts by Laing, Cooper, Minkowski, and Binswanger. Several other members of Psichiatria Democratica (Pirella, Piro, Slavich and Giacanelli) shared the phenomenological background, and their thinking was permeated by phenomenological concepts. Novello [15] refers to the critique of asylum psychiatry and to the practice of Husserl’s epoché, namely the bracketing of psychiatric diagnosis [21]. Phenomenology resonated in the group’s attempt to understand the ‘presence’ (or ‘esserci’, ‘Dasein’) of the patient as a person, often devoid of cultural instruments but capable of expressing a personal life-project.

Novello refers to Basaglia‘s *Scritti* [22] in discussing the role of the concept of the ‚body‘ in criticising institutional psychiatry. The ‘monstrous’ patient’s body coincides with the institutional body, a dangerous body ‘lived by’ the institution and ‘for’ the institution [15]. The phenomenological perspective is evident, and the practice of institutional psychiatry can be seen as controlling the patient’s ‘obscene body’ that ‘embodies’ social dangerousness [22]. The discourse about “obscenity” and Basaglia’s interpretation of community psychiatry “in a Straussian key” [22], preceded and followed correspondence between Basaglia and Erling Eng, an American psychologist, colleague of Straus and translator of part of his work who was active in the phenomenological circle of Lexington [letters dated March 5, 1966, December 23, 1966, and February 14, 1967 - BA, Serie 11 Corrispondenza]. Basaglia’s project moved toward a broader cultural and epistemological debate focusing on the analysis of the institutional ‘apparatus’ (dispositivo istituzionale) following Foucault and Deleuze and on the ‚language of the clinic’. However, Basaglia insisted on phenomenology as the foundation of his thinking and activism. It was Eugenio Borgna who considered Basaglia a pioneer of existential philosophy in post-war Italy (referring to Jaspers, Binswanger, Minkowski, and Straus; [14, 23, 24]). Basaglia corresponded with Minkowski, and the correspondence refers to at least one personal meeting [letters dated March 9, 1968, and February 11, 1968 - BA, Serie 11 Corrispondenza].

Novello refers to the concept of *Daseinsanalyse* and to a text by Basaglia of 1953 entitled ‚Il mondo dell’incomprensibile‘ schizofrenico attraverso la *Daseinsanalyse*‘ [15] criticizing Jaspers’ subjective phenomenology with reference to the concept of objective phenomenology by Husserl and Binswanger. Basaglia endorses Binswanger’s *Daseinanalyse* as having given meaning to psychotic experience by revealing, in listening to individual stories, the unique ways in which a person ‘has opened up to the world’. There is a clear influence of Eugène Minkowski’s thinking (lived time-space [25]). Texts of the mid-1960s document continuity with phenomenology.

Basaglia’s Gorizia work years (1961–1969) saw the project of institutional change and therapeutic community [26]. Basaglia highlights the risk of an ‘aestheticising being-in-the-world’ inherent in phenomenological approaches and emphasizes that persons with mental illness should be seen as part of their social world, beyond the syndromal framework of biomedical science. Novello argues that the mid-1960s reflect the passage from criticising psychiatric nosography and the patient-therapist-relationship to a wider anti-institutional struggle. Social exclusion of the mentally ill moves center stage. Basaglia considered phenomenology an answer to dehumanization and institutionalism cautioning that it might help only the wealthiest. Almost all asylum patients were members of the (sub-) proletariat: questions of power and inequality needed to be addressed [15, 26]. Basaglia, in response to criticism of the reform, points to the challenge posed by asylum living conditions criticizing the ‘cynical negation’ of mental illness expressed through the absence of care and support [15, 22]. He insists on the long-term nature of the reform involving politics, trade unions and social groups. Basaglia used the image of ‘sinking the ship’ (with reference to Sebastian Brant’s *Das Narrenschiff*, The Ship of Fools), as there could be no return to the asylum, an analogy referring to the explorer Hernán Cortés burning his expedition ships to convince his soldiers there was no way back [27]. With asylum reform and closure, patients had improved their contractual position, sharing in social activities outside the institution. The debate focused on the acquisition of contractual power, freedom of movement and social participation in community settings. Basaglia argued that people with mental illness (now in the open) would join other groups on the margins of society (e.g. those excluded from school and the labor market, those living precariously and with disabilities) not in possession of full civil rights.

### Jean-Paul Sartre

There is a towering presence of Jean-Paul Sartre’s work in Basaglia’s writings (and in the literature on Basaglia’s work). Sartre was a key proponent of philosophical existentialism, with a focus on individual freedom and responsibility [28]. It was clear to his colleagues that Basaglia considered Sartre his ‘maestro‘ [15]. His interest dated back to his being given Sartre’s collected works as a present on the occasion of his marriage to Franca Ongaro in 1953 [15, 29, 30]. References to Sartre, in Basaglia’s work, are often to ‘Critique of Dialectical Reason’ (CDR) [31] while ‘Being and Nothingness’ (BN) [32] is mentioned less. In his transition from BN to CDR, Sartre moved from phenomenological ontology via what he referred to as the ‘mediating third’, the ‘practico-inert, and ‘basic sociality’ to his notion of ‘dialectic and praxis’. Sartre, in CDR, introduced key terms such as need, transcendence, ‘project’, and ‘process’. The book examines the components of social ensembles, and against the background of Sartre’s emphasis on ‘scarcity’, the concept of ‘labour’ emerges. Flynn [33] describes Sartre as moving towards a ‘structural anthropology’, and social and group processes are acknowledged as central.

According to Basaglia’s correspondence, the first step in Basaglia’s interaction with Sartre was a letter from Minguzzi, dated October 13, 1966, [BA, Serie 11 Corrispondenza]. Numerous references to meetings that took place on various occasions exist: e.g., in the preserved draft of a letter to Cooper, dated August 16, 1968, Basaglia writes that he will meet Sartre in Paris at the end of September. In the correspondence with Castel various joint projects are mentioned in 1972 and 1973 [BA, Serie 11 Corrispondenza]. The books ‘Che cos’è la psichiatria?‘ and ‚Crimini di Pace‘ [34–36] reflect Sartre’s influence [15]. These books were published (and translated) in the 1970 s, focusing on the marginal societal position of people with mental illness, the power/knowledge nexus in the psychiatric profession, institutionalism, the ‘moral career’ of patients in the asylum, and the professional roles of psychiatrists as ‘technicians of practical knowledge’. CDR is referenced in ‘Che cos’è la psichiatria?‘, particularly in relation to the role of the ‘scientist’, the researcher’s distance, and professional roles in a coercive system. Gallio [15] describes the influence of the 1968 student movement on that year’s meeting between Sartre, Franca Ongaro, and Franco Basaglia. ‚Crimini di pace‘ [35] contains the transcript of a discussion with Sartre on the role of the intellectual as technician of practical knowledge. Basaglia argued that the closure of the psychiatric hospital must be accompanied by the search for alternative practices and a new science addressing the needs of people with mental illness. This new science could only be developed in partnership with service users [35].

Basaglia‘s texts of 1962–1967 contain many references to Sartre and the institutional apparatus (*dispositivo istituzionale*). In 1968, Basaglia’s focus shifted to a broader epistemological debate. People with mental illness were considered one group among a number of ‚subaltern groups’ or classes, e.g., people considered deviant, diverse, and dangerous, including migrants, sexual minorities, etc. That period reflected the passage of Basaglia’s theoretical position from BN to CDR [31, 32] and increasing reference to Foucault.

At the first International Congress of Social Psychiatry held in London in 1964, Basaglia had first articulated the programme of mental hospital closure, defining three axes of action:

1. a Sartrian ontology of freedom;
2. the critique of the devastating effects of institutionalisation; and.
3. the relationship that the psychiatrist needed to establish with professional knowledge and power.

Gallio suggests that it was Sartre’s radical humanism that Basaglia adopted as a foundation for his secular ethic and for his criticism of psychiatric institutions [15].

Following the culture clash between anti-communism and anti-clericalism in 1960 s Italy and amidst wider societal transformation from below, structural reform focused on citizen rights (including workers’ issues, divorce, abortion, schools, the health service, and mental health). In the practical reform struggle, despite the influence of Sartre and Basaglia’s contacts with French psychiatrists and theoreticians (see correspondence, Archivio Basaglia, Venice, Italy), Basaglia did not adopt the French models of ‚Psychiatrie de secteur‘ or ‚Psychothérapie institutionelle‘. In the debate on psychiatric reform, he opted for the analysis of ‚total institutions‘ by Goffman [37] and for Anglo-Saxon models, e.g. the American Community Mental Health Center and Maxwell Jones’ therapeutic community [38].

Gallio argues that Basaglia combined Anglo-Saxon impulses with Sartre’s radical humanism underlining people’s right to transformation ‚ciascuno della sua realtà‘ (each one of their reality) [15]. A Basaglia-Sartre meeting in Bologna (1968) was followed by other encounters [36, 39]. Sartre‘s interest in people suffering from schizophrenia was explicit. He contributed an introductory text to Frantz Fanon’s ‘The Wretched of the Earth’ [40] and to Laing and Cooper’s book ‚Reason and Violence‘. In an article published in Les Temps Modernes entitled ‚L’homme au magnétophone‘ [41], Sartre referred to psychiatric reformers in England and Italy as seeing, in each patient, the subject endowed with the freedom to act. In a 1963 lecture in Rome ‚Ansia e malafede’ (Anxiety and bad faith), Basaglia quoted Sartre in the epigraph stressing that healing (by psychiatrists) and recovery (by patients) were impossible in the absence of ‚freedom’ and relationships of reciprocity. According to Gallio [15], it was in texts of 1962–1966 that Basaglia struggled with Jaspers’ approach and found his philosophical masters in Husserl and Sartre. It was in connection with Husserl’s method of managing ignorance or prejudice through ‘phenomenological epoché’, i.e. the bracketing of psychiatric diagnosis (during a phase of radical transformation) that Basaglia concluded that the asylum, as a social ensemble, could be ‘destroyed’.

Basaglia drew a line from phenomenology (Minkowski, Binswanger, Zutt) to Sartre. From the mid-1960s, Sartre’s thinking and activism moved to the centre of his thinking. And there was a lot of resonance with the Sartre of CDR in the day-to-day work of institutional reform. Meetings of the Gorizia therapeutic community constituted moments of self-reflection. The social roles of patient and psychiatrist were questioned. The work of the therapeutic community was about moments of choice in practical day-to-day living discussed during meetings. This practice produced series of day-to-day micro-events documenting the potential for change.

### Michel Foucault

Foucault is referred to as a key poststructuralist European thinker. His intellectual itinerary can be described as evolving from early influences of hermeneutics and phenomenology towards the development of an archaeology and, later, a ‘genealogy’, understood as a methodological framework to analyze systems of power and knowledge. He dealt with knowledge systems such as psychology, psychiatry, biology, economics, and linguistics. His work aimed to uncover the epistemic assumptions underlying complex thought systems, with particular attention to ‘discourse’ conceived as the process of constructing reality. One of his biographers argued that Foucault’s relativism undermined the utility of his theories [42]. His thinking holds a key place in modern critical thought. He was open to political activism [43, 44]. Foucault, in ‘Madness and Civilization’, presented a social history of the construction of madness in Western societies. His approach was distinctly relativistic framing madness as a cultural, legal, political, philosophical and medical construct [45]. Foucault’s primary focus was on the post-Enlightenment period [46–48].

Foucault was one of the authors of ‚Crimini di pace‘ [35]. In his contribution, he introduces the concept of ‘technologies of truth‘, outlining a historical chronology of places in which truth was produced up to the 18th century. Prior to that period, truth was not conceived in terms of “what is or happens”, i.e. as an event. It was not ascertained, but aroused: “production instead of revelation” [35], not discovered through instruments, but produced through rituals, e.g. in alchemy. Foucault identifies a shift from ‘truth through the application of procedures’ to truth as ‘ascertainment’. In the late 18th century, experimentation generated ‘verifiable truth’, and modern science emerged. The transformation of knowledge procedures was accompanied by socioeconomic change and by institutionalisation of power-knowledge relationships (knowledge as power, societal elites). Diseases began to be ‘observed’ in hospitals, and the hospital became a place of diagnosis, clinical identification, experimentation and treatment.

Before the 18th century there had been no specific interest in the systematic treatment of people with mental illness. Foucault describes the French 19th century alienist approach in dealing with deviance (in the asylum) [46]. Truth is ‘produced’ through the power of the doctor. The procedures implemented in mental hospitals, such as isolation, interrogation, treatment-punishment, rigorous discipline, but also confidentiality, relationships of domination and trust, tended to make the doctor the master of madness. This approach entered into crisis after Charcot. Foucault describes the subsequent development of psychiatry by identifying two main lines: medicalisation of madness and antipsychiatry.

During the ‚Conferenze Brasiliane‘, Basaglia described a profound transformation of selves among psychiatric reform activists in the course of the Italian reform, an ethical and spiritual experience along a dimension of ‚truth‘ that resonated with Foucault’s teaching in his 1982 lecture at the Collège de France on ‚The hermeneutics of the subject‘ [16, 49]. Colucci discusses Basaglia’s ambivalence toward theorization and his prioritizing of institutional transformation. One of the debates (in Brazil) was called ‚Technicians or intellectuals?‘. Sartre’s concept of ‚technicians of practical knowledge‘ resonated with Foucault’s notion of the ‚specific intellectual‘.

According to Basaglia, dismantling the link between knowledge and power is extremely challenging. Yet mental hospital closure and striving towards alternative mental health care practice can help. Colucci [12] highlights two core elements in Basaglia’s praxis: first, always concentrating on detail, working with specific challenges based on patients‘ needs and refraining from abstraction; second, stepping outside the specifics of psychiatry and moving to the wider field of institutional power (as described by Foucault). Practical reform may require professional sacrifice, such as the loss of the expert role and of institutional structural clarity. Foucault’s broader philosophical project with its focus on the historical and epistemic struggle between freedom and control and knowledge and power, closely paralleled Basaglia’s theoretical orientation and his struggle in closing the asylum and concentrating on patient rights [44].

## Discussion

The Italian psychiatric reform, and Basaglia’s and his team’s work in transforming mental health care in Italy, have both attracted international attention [50]. The process showed parallels and differences with other European countries, e.g. in the closure of mental hospitals (paralleled in Great Britain, not paralleled in France and Germany), and in the construction of a nation-wide system of community mental health care (paralleled in Britain). In order to understand mental health reform processes, a range of socioeconomic, national political and administrative factors must be considered. An important role is also played by theoretical and cultural frameworks, including philosophical and social science contributions [51, 52]. Among the most influential theoretical contributions to European psychiatric reform, there was Goffman’s seminal book ‘Asylums [37]. Other important references included the work of Laing and Cooper (associated with antipsychiatry), and thinkers such as Jaspers, Husserl, Minkowski, Binswanger, Foucault, Sartre and Gramsci.

Basaglia’s and his colleagues’ writings constitute an impressive body of theoretical reflection with recurring themes. Despite limitations in bibliographic documentation (and a lack of systematic compilation and editing of texts), the background of Basaglia in phenomenological philosophy and psychiatric phenomenology is clear, and its foundational role can be seen evolving from the central concept of the ‚body‘. In Basaglia’s social philosophy, Sartre’s books BN [32] and CDR [31] are core references. A text that epitomises a central driving force of Basaglia is the essay ‘Corpo, sguardo e silenzio’ [53] which questions the enigma of subjectivity in psychiatry. The theoretical core of the reflection lies in the concept of ‘distance’ between the subject and his/her body that must be calibrated and qualified. Drawing on key phenomenological thinkers, from Husserl to Merleau-Ponty, Basaglia places emphasis on Sartre’s reflection on corporeality and the gaze in the relationship with the ‘Other’. Basaglia analyses some texts of Italian playwright and novelist Pirandello. His interest in Pirandello is likely to reflect his interest in human freedom, constantly interpreted as a relationship between normality and madness - the phenomenological core of Pirandello’s poetics and philosophical outlook [54].

According to Webber [28], Sartre’s thinking builds on the idea that „an individual’s character consists in the projects that person pursues“ [28] emphasising radical freedom with human beings ‘condemned to be free‘. Sartre’s phenomenology begins with the assertion „that a person is a body, a physical object, with consciousness“ [55]. His „concept of the mind is formed independently of, and serves as a basis for the concept of the person” emerging by combining the concepts of mind and body.“ [55]. Thus, the concepts of ‚body and lived experience’ both hold a central place in Sartre’s phenomenology and are likely to have contributed to Basaglia’s intellectual affinity. The importance of the Other, of the Other’s look and of being-with-the-Other as constituting personhood also resonates strongly with Basaglia’s interest in encounter as the central element of helping relationships.

In a book by Stack [56], we find the unusual notion of „the body as coextensive with the world [as] the foundation of man’s being-in-the-world“ - a notion that is present in both Sartre’s and Basaglia’s thinking [56]. As Sartre explores the social conditions of social existence, he „fully realizes that his phenomenology of concrete, free action is a double-edged sword” as humans inevitably encounter limitations which are immanent in „the given [world] and which it [freedom] endeavors to surpass.“ [56]. Stack emphasises continuity between the earlier ontology of BN and the later social phenomenology of CDR. As Sartre admits, „I encounter the other in a social milieu, in a world already constituted by systems of meanings I have not created.“ [56]. His central theme is the dialectical tension between individual freedom and the deterministic structures of social milieus [56]. Sartre speaks of ensembles, and he reveals a ‚lived reciprocity‘ which is the basic component of social existence [56].

It is Foucault who contributes the ‘interpretive analytics’ element [57] and who shares Basaglia’s and Sartre’s profound interest in the ‘body’. In the opening chapter of ‘Discipline and Punish’ entitled “The Body of the Condemned”, Foucault declares his aim to write “a history of the present”. This implies the analysis of knowledge systems by delineating their historical origins, and using genealogy to reconstruct how ‘bodies’, particularly those of mad people or those in marginal positions, are shaped. Foucault’s relativistic approach, his archaeology and genealogy go hand in hand with political activism. In Basaglia’s work we discern both phenomenological thinking and a philosophy of social existence as points of reference, with weight given to group processes, the interaction with the ‘Other’ and the necessity to struggle against exclusion and segregation. The resonance with Foucault’s interpretive analytics and Sartre’s social philosophy is impressive. This convergence is likely to have helped Basaglia develop his leadership integrating theoretical discourse with the practical language of the therapeutic community and anti-institutional reform.

Basaglia‘s writings show the signs of their time: the dialogue with phenomenology, existentialism and a socio-centric social philosophy. His commitment to practical psychiatric reform and his reluctance to engage in theorization echo the activist commitments of both Sartre and Foucault. However, there is another, highly original contribution we should acknowledge: over the course of a two-decade anti-institutional struggle, Basaglia developed a highly specific approach, close to Gramsci’s ‚philosophy of praxis‘ [58].

### Limitations

Our review has several limitations. First, the literature reading on which this review is based was selective. Although a multitude of sources were used, there is a risk of biased perception and distortion owed to the selection of sources. Second, the dynamic of interactions between theory and practice, in the 1960 s and 1970 s, was entirely different, amidst the struggle for psychiatric reform and in the context of wider societal movements and debates in Italian society, from the context in which this narrative review attempts to understand past interactions and cross-references today, with inevitable hindsight bias. This difference in the social and cultural dynamics could have led to a misunderstanding of interactions as they evolved at the time suggesting a cautious interpretation of the findings. Third, this article concentrates on the professional perspective of the medical-psychiatric profession. This may have led to an under-representation of contributions by members of other mental health professions, such as nursing, social work and psychology, in the debates of the 1960 s and 70s.

## Conclusion

Basaglia’s anti-institutional reform work (and the wider politico-cultural situation in Italy) resonated with some of the thinking of 20th century philosophers Sartre and Foucault. There can be little surprise that Husserl’s approach in abstaining from ‘conclusion or judgement’ suited Basaglia and his colleagues in their project of setting aside some of the clinical issues in the face of dramatic institutional neglect and deprivation. Basaglia was aware of the risks involved. There is further resonance, in this unique post-Fascist anti-institutional reform project, with the philosophy of Friedrich Nietzsche who was a key reference for both Sartre and Foucault. We can intuit that Nietzsche’s anti-Hegelian thinking and its destructive potential might have been helpful in confronting the apocalyptic reality of provincial mental hospitals in Italy in the 1960s. The project was built pragmatically while the theoretical framework was able to cope – metaphorically – with the ‘destroyed body’, the reality of ‘madness’, the totality of the institution, and the urgent need for a new language that would help, culturally, to decipher human suffering and build therapeutic projects. It is impressive to see how, in the very late text of ‘La vocazione terapeutica’, Basaglia and Gallio [38] focused on the therapeutic imperative and the search for a ‘therapeutic language’ in a mental health service operating in new surroundings (within a changing society).

Basaglia’s thinking was a pointer to the future in considering service user perspectives [59–62] and the issue of epistemic justice [63]. Basaglia was more than two decades ahead of his time in identifying the central importance of the contribution of people with first-person experience of mental illness [64] in defining peer support, fighting institutionalism and achieving mental health reform. It is for this reason that papers addressing the promise phenomenology holds for person-centered psychiatric care in our time refer to and concentrate on Franco Basaglia’s work [18].

One of the reasons for Basaglia’s continued relevance may lie in the knowledge-power link that Michel Foucault delineated in ‘Madness and Civilization’. The institutional dilemmas of psychiatry and mental health care have been analyzed and discussed with excellent science and impressive civil society debates. However, where should ‘meta-community psychiatry’ [65] be moving today, and what theoretical considerations would help generate the reform enthusiasm that fueled much of the reform movements of the last quarter of the 20th century? There is, currently, an ongoing (and renewed) debate in the field. While substantial progress has been made in better defining the recovery concept [66], and while recovery colleges constitute a strong structural basis for the dissemination of recovery-oriented care [67], some authors have made critical contributions. In a scoping review of the concept of co-production, Norton [8] points to a “philosophical war between co-production and that of evidence-based practice itself” (reflecting the contrast between evidence-based medicine within an empiricist-enlightenment perspective and constructivist approaches as prevalent in the user and survivor movement and mad studies). Rose and Kalathil [9] argue that co-production between researchers, policy makers and people “positioned as mad, particularly as mad people of color, cannot happen in knowledge production environments operating within assumptions and philosophies that privilege reason as well as white, Eurocentric thinking.” Caution is also expressed in work on recovery colleges, where the authors emphasize that “a certain distance is needed in the relationship between the Recovery College and its host organization” [67]. Armed with the social science and philosophical armamentarium as provided by Husserl, Sartre, Foucault and others, Franco Basaglia recognized these contradictions, and his grappling with the issue is evident in the utopian, unfinished element of his search for a ‘new mental health care practice of reciprocity’ beyond traditional biomedical patterns.

What theories should we turn to in our search for modern mental health services today? The authors suggest that it is a radical focus on ‘dialogue’ or ‘dialogic mental health practice’ that we should call for as mental health professionals (and citizens). As in the 1960 s and 70 s, there may be some loss to the status and economic-institutional power of the psychiatric profession along the way, but the effort may benefit people with severe mental illness (and wider society). The effort should be combined with renewed interest in issues such as those raised by Nobel Memorial Laureate (in Economic Sciences) Amartya Sen who discussed ‘Collective choice and social welfare’ [68] and the economic issues of non-market economies pursued by Economic Sciences Nobel laureate Elinor Ostrom in her ground-breaking work on institutional diversity and common goods [69]. As Norton [8] points out, it was Ostrom who first coined the concept of ‘co-production’ in the 1970s. Taking this direction might help us devise social spaces capable to deal with the precarious states shared by many. It may be an effort similar to the theoretical search Franco Basaglia pursued in the 1960s and 70 s that will help us shed more light on the conundrum of mental illness today. We need to remember his quest, of five decades ago, in our search for a dialogic citizen psychiatry in the context of a broader societal welfare approach.

This approach could benefit from making use of the experiences made by marginal communities around the globe in coping with their problems, e.g. homelessness, hunger, or financial distress. Appadurai [70] describes the work of the Mumbai Alliance, dating back to the 1980 s, in fighting the key problems of slum dwellers in metropolitan Mumbai, i.e. homelessness, financial distress, and lack of toilets. The Alliance’s grassroots approach involved negotiation, consensus, daily (micro-) savings, problem-centered collaboration with relevant authorities irrespective of political orientation (politics without parties), horizontal organization, self-surveys (data collection), precedent setting, and exhibitions showing what has been achieved in the areas of women’s daily savings, housing, and the provision of toilets. Networking is essential and extends globally (Slum Dwellers International). Against this background, Becker et al. [71] argue that an active redistribution and allocation of resources and responsibilities to user-controlled services and organizations should be considered. These organizations would not exclusively focus on mental ill health, but cater for the needs of marginal groups more broadly, comprising issues such as homelessness, financial distress, substance use and other mental health problems. This project would embed (some of) the services available for people with mental health problems in a wider social arena, it would encompass co-production and recovery work, and such user-controlled services and the mental health care system would ideally seek close collaboration at eye level.

## Data Availability

No datasets were generated or analysed during the current study.
